# Performance Analysis of Metalenses Based on Three Kinds of Phase Compensation Techniques

**DOI:** 10.3390/nano11082091

**Published:** 2021-08-18

**Authors:** Peiyao Lu, Changda Zhou, Zhen Mou, Danhua Liu, Shuyun Teng

**Affiliations:** Shandong Provincial Engineering and Technical Center of Light Manipulations & Shandong Provincial Key Laboratory of Optics and Photonic Device, School of Physics and Electronics, Shandong Normal University, Jinan 250014, China; 2020020564@stu.sdnu.edu.cn (P.L.); 2019020514@stu.sdnu.edu.cn (C.Z.); 2018020551@stu.sdnu.edu.cn (Z.M.)

**Keywords:** metasurface, focusing metalens, vortex metalens, phase compensation

## Abstract

The phase delays introduced by anisotropic nanounits include propagation phase delay, resonant phase delay and geometric phase delay. Various phase devices can be formed based on the metasurfaces consisting of anisotropic nanounits and the phase devices of the same kind function have different performances because of different working modes. In this paper, metalenses and vortex metalenses are chosen as examples to compare the optical performance of metasurface phase devices based on three kinds of phase compensation techniques. We design separately three kinds of metalenses and vortex metalenses using the cross nanoholes, L-shaped nanohole and V-shaped nanoholes and simulate numerically their intensity and phase distributions. Additionally, the results show the differences among these elements in structure complexity, polarization dependence, working efficiency and phase uniformity. The comparison for three kinds of metalenses clearly shows the merits of different phase compensation techniques and this work must be helpful for expanding the practical applications of metasurfaces.

## 1. Introduction

Phase is the main property of light fields and it even determines amplitude, polarization state or other properties of light field. Phase of light field is usually modified by the phase element through the diffraction effect of element or refraction effect of material, like the wave plate, Soleil-Babinet compensator and Fresnel lens. 

Besides these traditional techniques, recent studies show the phase delay can be con-trolled by the nanometer units, such as subwavelength gratings [1,2], nanoantennas [3], nanoholes [4,5]. Metasurfaces consisting of nanometer units can introduce abrupt and dis-continuous phases so as to achieve complete control of phase, amplitude and polarization state of optical field [6,7,8,9]. The introduced phase by metasurface unit can be adjusted through changing the size, the shape and the rotation angle of nanounit. This phase can be differentiated into propagation phase relating to the width of nanounit, resonant phase changing with the length of nanounit and geometric phase depending on the rotation angle of nanounit in terms of the phase modulation mode [10].

Thus, metasurfaces have been proposed to control the phase and wavefront of light field, and various kinds of the metasurface elements including metalens [11,12,13], metasur-face hologram [14,15], beam deflector [16,17], vortex generator [18,19,20] and vector light generator [21,22,23] have been advanced. The designed metasurface elements based on different working modes have different optical performance. Resonant phases caused by nanoantennas with different shapes and different lengths are utilized in the design of the beam deflector [24] and the non-diffraction beam generators [25]. The high working efficiency is obtained just because of the resonant interaction of light and nanounits. Propagation phases caused by nanounits with different widths are utilized in the design of high-efficiency Bessel beam [26] and metalens [27]. These two kinds of metasurface devices show the polarization independence. Geometric phases caused by the rotated nanoscatterers are also utilized in the design of Bessel beams [28] and metalens [29,30]. With comparison to two former cases, the phase elements based on geometric phase have the polarization dependence. The difference between the phase elements with the same function and different working modes is far more than that, and yet the performance of these phase elements has not been compared till now.

This paper aims to construct the phase devices based on three kinds of phase com-pensation techniques and exhibit the advantages of different techniques through comparing optical performance of these devices. The metalenses and vortex metalenses are chosen as the metasurface devices to show the merits of different phase compensation techniques, where the nanometer units take the nanoholes with different shapes. V-shaped nanoholes with different length and cross angle are used as the resonant units, L-shaped nanoholes with different length and width are chosen as the propagation units, and cross nanoholes with different rotation angles are used as the geometric units. The parameters for nanounits are given in Section 2. The comparison of the performances of the designed devices is detailed in Section 3 and Section 4. The further discussions are given in Section 5. In the end, the conclusions of this paper are given. 

## 2. Determination for the Nanounit Parameters

As we know, the controllable phase should cover the values from 0 to 2π so as to construct one phase element. Here, we choose V-shaped nanoholes, L-shaped nanoholes and cross nanoholes to introduce respectively the resonant phase, the propagation phase and the geometric phase and construct the metalenses and vortex metalenses. Therefore, each kind of nanohole needs to introduce the phase values from 0 to 2π and the determination for nanounit parameters is the key to design the metasurface device.

**Figure 1:**
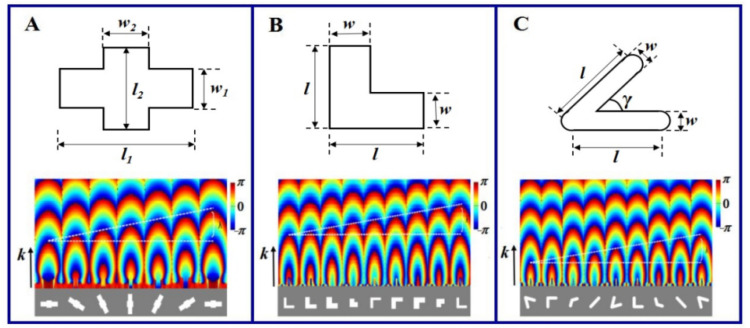
(**A**) Rotated cross nanoholes, (**B**) eight L-shaped nanoholes and (**C**) eight V-shaped nanoholes and their transmission phase distributions.

As we know, one rotated anisotropic unit can introduce the geometric phase, like the rotated cross nanohole [29]. Figure 1A shows the structure of cross nanohole, which can be taken as the combination of two rectangular holes with two lengths of *l*_1_ and *l*_2_ and two widths of *w*_1_ and *w*_2_. Since the additional phase of the rotated cross nanohole always appears among the cross circular polarization state, we should optimize these parameters of cross nanohole so as to ensure the maximum polarization conversion efficiency. Through our simulations, the thickness of silver film takes 220 nm, and two lengths and two widths take *l*_1_ = 600 nm, *l*_2_ = 220 nm, *w*_1_ = 150 nm and *w*_2_ = 180 nm. Here, the cross nanohole can be equivalent to one half wave plate and it can convert the circular polarization into the cross polarization with 100% conversion efficiency. The transmission phase covering the region from 0 to 2π can be obtained through rotating the cross nanohole, and the color patterns in the lower of Figure 1A give the transmission phase distributions of the single cross nanohole with the rotation angle taking different values. One can see that when the cross nanohole rotates from 0 to π, the transmission phase uniformly changes from 0 to 2π. Thus, the rotated cross nanoholes can construct the phase element through introducing the geometric phases.

The L-shaped nanohole can introduce the propagation phase because of the equivalent wave-guide effect [31]. Figure 1B shows the structure of L-shaped nanohole, where two orthogonal arms have the same length and width. In order to obtain the transmission phase covering the region from 0 to 2π, we also simulate the transmission of L-shaped nanohole with the arm length and arm width taking different values. Then we select four L-shaped nanoholes with the arm length and width taking 240 nm and 60 nm, 260 nm and 120 nm, 260 nm and 190 nm, 190 nm and 130 nm since these four nanoholes can introduce the phases with equal separation from 0 to π. The other phases from π to 2π can be obtained through rotating these four nanoholes by π/2. The transmission phase distributions of eight L-shaped nanoholes are shown in lower of Figure 1B. One can see that the phases transmitting from these eight L-shaped nanoholes uniformly change within the region of [0, 2π]. Therefore, these eight L-shaped nanoholes can construct the phase device through introducing the propagation phases.

For the V-shaped nanohole, as shown in Figure 1C, the parameters including the arm length, arm width and cross angle of two arms need to be determined in order to obtain the different resonant phase delays [25]. Among the simulated results, we select four V-shaped nanoholes with the arm width set at 40 nm and the arm length taking 158 nm, 148 nm, 110 nm and 113 nm. These four nanoholes can introduce the additional phases with equal separation from 0 to π, and the phases of π to 2π can be obtained through rotating these four nanoholes by π/2. The transmission phase distributions of these eight V-shaped nanoholes shown in the bottom half of Figure 1C verify the controlled phase covering the region from 0 to 2π. Therefore, these eight V-shaped nanoholes can be utilized to design the phase elements such as metalens and vortex metalens.

It needs to be pointed out that during the simulations, all the nanoholes are etched on the silver film and the silver film is deposited on the glass substrate. The thickness of silver film is set at 220 nm for consistency. The incident wavelength is set at 633 nm and the illuminating light impinges on the nanohole from the glass. The simulation calculations for the transmission phase distributions of nanoholes are performed in terms of the finite-difference time-domain (FDTD) method [32]. The calculation region for one single nanohole is 2 μm × 2 μm × 5 μm. The tested plane is set at xoz plane. The perfectly matched layers are used to prevent non-physical scattering at the calculation boundaries. The mini-mum mesh step is set at 2 nm to ensure the calculation precision. The dielectric constant of the silver film takes the values given by Palik E D [33].

## 3. Metalenses Based on Three Phase Compensation Techniques

As we know, for the metalens consisting of nanounits, the phase delay of the nanounit with the radial coordinate of *r* should satisfy the following form [34],

(1) $$\varphi =\frac{2\pi }{\lambda }[\sqrt{r^{2}+f^{2}}-f]$$

where *f* represents the focal length of metalens and *λ* is the illumination wavelength. Among the geometric metalens consisting of cross nanoholes, the nanoholes can be located at any radial position. In terms of the relation that the phase delay is twice of the rotation angle *θ*, the rotation angle of cross nanohole at the radial coordinate of *r* equals to half of the above phase delay. For the L-shaped nanoholes, they only introduce eight phase delays with the equal separation, and we can denote the transmission phase delays as *φ_i_* with *i* taking the number from 1 to 8. From Equation (1), one can determine the position of the *i*th V-shaped nanohole as

(2) $$r_{i_{n}}=\sqrt{{[(\frac{\varphi_{i}}{2\pi }+n)\lambda +f]}^{2}-f^{2}}$$

where *n* takes any integer. Obviously, for a different L-shaped nanohole, its radial coordinate is different. Similarly, for the V-shaped nanoholes, the position coordinates of eight L-shaped nanoholes can be determined according to Equation (2). Thus, we design the metalenses with the same focal length of *f* = 4.5 μm and the same numerical aperture based on three kinds of nanoholes and parts of the structure parameters for metalenses are shown in Table 1. For three kinds of metalenses, the nanoholes at the same radius are the same, yet the nanoholes at the different radius have different parameters.

**Figure 2:**
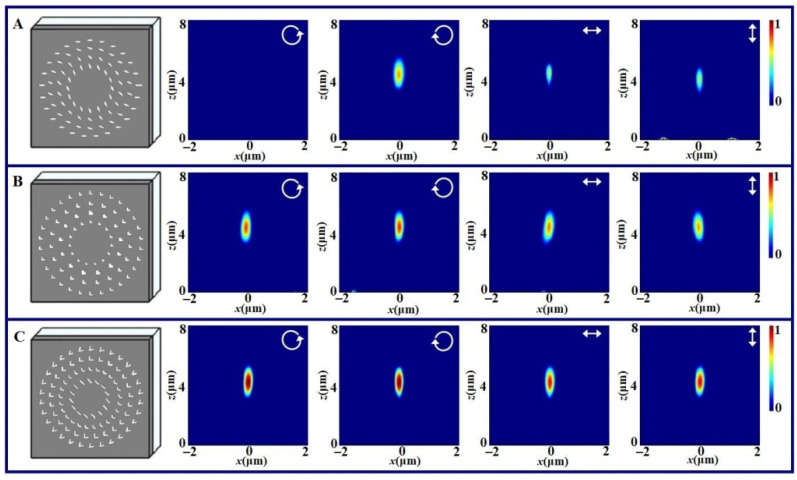
Schematic diagrams of three metalenses and their focusing intensity distributions with LCP, RCP, HLP and VLP light illumination, where (**A**) is for the geometric phase metalens, (**B**) is for the propagation phase metalens and (**C**) is for the resonant phase metalens. The inserted arrows in the intensity distributions denote the incident polarization states.

In order to ensure the same transmittance, the total transmission area of nanoholes for each metalens is the same, and the transmission area of all the nanoholes at the same radius also takes the same value. Figure 2 gives the schematic diagrams for three kinds of metalenses and their focusing intensity distributions with the left-handed circularly polarized (LCP) light, right-handed circularly polarized (RCP) light, horizontal linearly polarized (HLP) light and vertical linearly polarized (VLP) light illumination. Figure 2A shows the results for the geometric phase metalens, Figure 2B gives the results for the propagation phase metalens and Figure 2C corresponds to the resonant phase metalens. For convenience comparison with respect to the intensity of the patterns in Figure 2C, the intensities in Figure 2A increase six times and the intensities in Figure 2B increase about three times. For all the structures, the nanoholes with the same parameters are on the same circles and the gap of two circles is about 1000 nm. 

From the results in Figure 2A, one can see that for the geometric phase metalens, the focusing shows the obvious polarization dependence. The focusing spot appears under the RCP light illumination and the focusing spot disappears under the LCP light illumination. With the HLP and VLP light illumination, the focusing spots take on with weaker intensity. This is because one linearly polarized light can be taken as the superposition of the RCP and LCP light and only the RCP component can be focused. The focusing with the polarization dependence may provide the polarization selection function for the integrated and multiplexing device.

From the results of Figure 2B, one can see that for the propagation phase metalens, the focusing always appears with any polarized light illumination though the intensity of the linear polarized light decreases slightly. Thus, the focusing effect shows the polarization independence. Similarly, the results for the resonant phase metalens shown in Figure 2C take on the similar focusing phenomenon. This characteristic of the polarization independence brings convenience for the applications of focusing metalens.

Comparing the focusing efficiencies of three kinds of metalenses, one can see that the peak intensity for the resonant phase metalens is the maximum and the peak intensity for the geometric phase metalens is the minimum. We extract the relative peak intensities of three metalenses with RCP light illumination from the simulated results, and the ratio of the peak intensities of three metalenses with RCP light illumination is about 1:3.08:6.81. Therefore, the polarization dependence and the focusing efficiency are the major difference for three kinds of metalenses. From the design process of different metalens, we can see that the structure arrangements of the resonant and propagation metasurfaces consisting of the V-shaped or L-shaped nonaholes are slightly complex than that of the geometric metasurface consisting of cross nanoholes because the different nanoholes need to be ar-ranged separately. Yet, the structure arrangements of three metasurfaces are easy to real-ize.

## 4. Vortex Metalenses Based on Three Phase Compensation Techniques

For the focusing vortex metalens consisting of nanounits, the transmission phase delay of any nanounit at the radial and azimuthal coordinate of (*r*, *α*) should satisfy the following form,

(3) $$\varphi =\frac{2\pi }{\lambda }[\sqrt{r^{2}+f^{2}}-f]\;+\;l\alpha$$

where *f* represents the focal length of vortex metalens and *l* takes any integer, which corresponds to the topological charge of vortex. Similar to the design of the focusing metalens, the nanohole of the geometric metasurface at any position needs to be rotated by the angle of *φ*/2. However, for the propagation and resonant metasurfaces, the V-shaped or L-shaped nonaholes at different radial or azimuthal positions are different. Along the same azimuthal angle, the radial position of the *i*th nanohole should satisfy

(4) $$r_{i_{n}}=\sqrt{[(\frac{\varphi_{i}-l\alpha }{2\pi }+n)\lambda +f{]}^{2}-f^{2}}$$

where *n* takes any integer. Evidently, the V-shaped or L-shaped nanoholes with different parameters appear at different radial coordinates along the same azimuthal direction. Reversely, the nanoholes at the same radius change with the azimuthal angle. Therefore, the structure arrangements of the resonant and propagation metasurfaces consisting of the V-shaped or L-shaped nanoholes is more complex than that of the geometric metasurface consisting of cross nanoholes.

**Figure 3:**
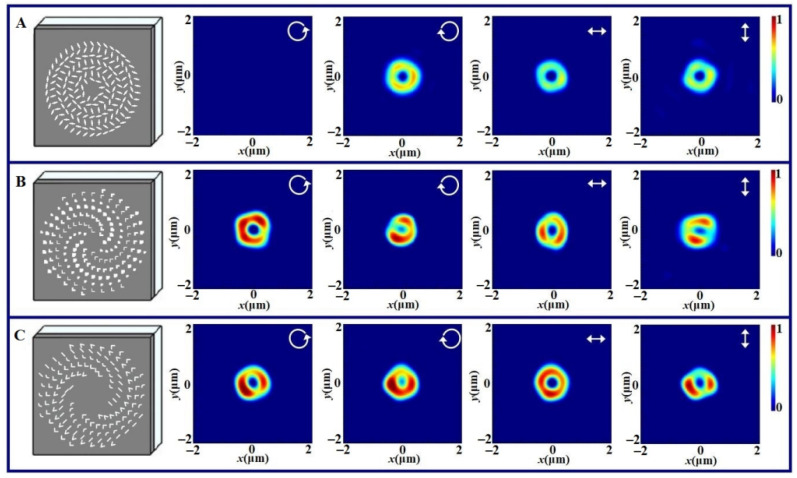
Schematic diagrams of three vortex metalenses with the topological charge of vortex taking 1 and their focusing intensity distributions with RCP, LCP, HLP and VLP light illumination, where (**A**) is for the geometric phase vortex metalens, (**B**) is for the propagation phase vortex metalens and (**C**) is for the resonant phase vortex metalens. The inserted arrows in the intensity distributions denote the incident polarization states.

Figure 3 shows the schematic diagrams of three vortex metalenses with *l* = 1 and their focusing intensity distributions with the RCP, LCP, HLP and VLP light illumination. Here, the incident wavelength still takes 633 nm. Figure 3A shows the results for the geometric phase vortex metalens, Figure 3B gives the results for the propagation phase vortex metalens and Figure 3C is for the resonant phase vortex metalens. Comparing the structures for three vortex metalenses, one can find that they are obviously different. The cross nanoholes for the geometric phase vortex metalens are arranged at the concentric rings and the rotation angle of nanohole changes with its radial and azimuthal coordinates. The nanoholes with the same shape among two latter vortex metalenses seem to be arranged along one spiral trajectory. In fact, the nanoholes on one circle have different parameters so as to form the spiral phase and the gap of two circles take about 800 nm. 

The structures of three vortex metalenses are consistent with the prediction of Equations (3) and (4). From Figure 3A, one can see that the annular intensity does not appear under LCP light illumination, but it takes on under RCP light illumination. The polarization dependence of the focusing vortex metalens is similar to the geometric metalens. Moreover, the focusing vortex has weaker intensity under the HLP or VLP illumination. This can be also explained by the point that the linear polarization contains the RCP and LCP components and only the RCP component can be focused. The annular intensity distributions of vortices for three latter cases are uniform.

The results in Figure 3B show that the annular intensity always appears under any polarized light illumination and the generated focusing vortex is polarization dependence. Similarly, the results in Figure 3C show that the focusing vortex is always generated and it is also polarization-dependent. However, the annular intensity distributions of vortices for two latter cases are not uniform. Comparing the intensities for three cases, we can find that the intensity of the resonant phase vortex metalens is the maximum.

From the shapes of vortices generated by three vortex metalenses, one can see that the vortex generated by the geometric phase vortex metalens is more uniform than those generated by the propagation phase vortex metalens and the resonant phase vortex metalens. Therefore, with the help of the focusing vortex generation, we can see that the differences for three kinds of vortex metalenses are reflected by the complexity of the structure arrangement, the polarization dependence, the focusing efficiency and the uniformity of vortex.

## 5. Discussions

**Figure 4:**
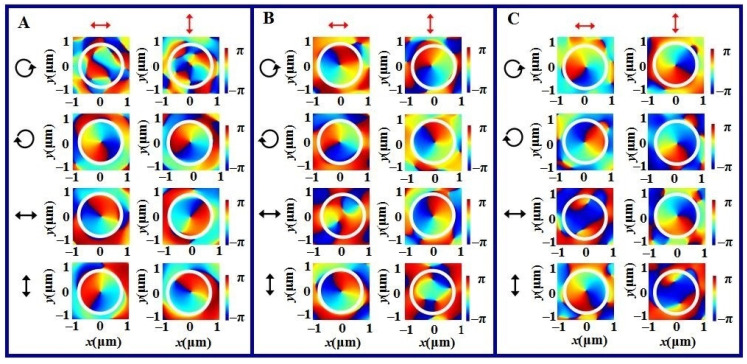
Transverse phase distributions of three vortex metalenses at the focal plane under different polarized light illumination, where (**A**) refers to the geometric phase vortex metalens, (**B**) is for the propagation phase vortex metalens and (**C**) corresponds to the resonant phase vortex metalens. The inserted black arrows on the left of patterns denote the incident polarization states and the red arrows on the top denote the detection direction of the polarization analyzer. The circles inserted in the patterns are convenient for the comparison.

Through comparing the focusing effect in Section 3 and Section 4, we know the focusing intensities are different for three kinds of metasurfaces. In order to further understand the phase difference for the elements designed in terms of different phase modulation modes, here, we concentrate on discussing the phase distributions for three vortex metalenses, and the structures of three vortex metalenses are the same as the ones in Figure 3. Figure 4 gives the phase distributions at the focusing planes of three kinds of vortex metalenses, where the incident polarization state takes RCP, LCP, HLP and VLP. Figure 4A shows the phase distributions of the geometric phase vortex metalens, Figure 4B corresponds to the phase distributions of the propagation phase vortex metalens and Figure 4C shows the phase distributions of the resonant phase vortex metalens. The black arrows inserted on the left sides of the phase patterns denote the incident polarization, and the red arrows on the top denote the detection direction of the polarization analyzer.

From the results given in Figure 4, we can see the phase distributions for different vortex metalenses are different. For the geometric phase vortex metalens, the phases for the x component and y component increase 2π along the anticlockwise direction with the LCP, HLP and VLP light illumination expect for the case with RCP light illumination, like the results shown in Figure 4A. This indicates that the topological charge of vortex is equal to 1 and it is the same as the prediction. The initial phase difference of the x component and y component is about π/2, and thus, the output vortex is always the RCP.

The results in Figure 4B show that the phases for the x component and y component increase by 2π along the anticlockwise direction with the LCP and RCP light illumination. The initial phase difference of the x component and y component is about π/2 for the former and –π/2 for the latter, and it means that the output vortex takes the cross circular polarization. Moreover, only the phase for the y component increases 2π along the anti-clockwise direction with the HLP light illumination and only the phase for the x component increases 2π along the anticlockwise direction with the VLP light illumination. This indicates that the vortex takes the orthogonal polarization. The similar phenomena ap-pear among the focusing phase distributions of the resonant vortex metalens.

**Figure 5:**
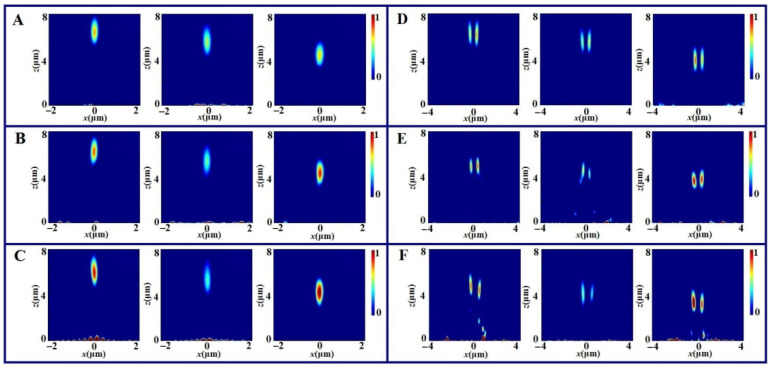
(**A**–**C**) Focusing of three metalenses with the illuminating wavelength taking 488 nm, 532 nm and 633 nm and the polarization taking RCP; (**D**–**F**) focusing vortex metalens with the illuminating wavelength taking 488 nm, 532 nm and 633 nm and the polarization taking linearly polarization along the x direction. Where (**A**,**D**) are for the geometric phase elements consisting of cross nanoholes, (**B**,**E**) are for the propagation phase elements consisting of L-shaped nanoholes and (**C**,**F**) are for the resonant phase ones consisting of V-shaped nanoholes.

Furthermore, for the metasurface devices, the dispersion effect also attracts much attention. We also numerically calculate the focusing effect of three kinds of focusing metalenses and focusing vortex metalenses with the illuminating wavelength taking different values, and the simulated results are shown in Figure 5. Figure 5A–C give the longitudinal intensity distributions generated by the focusing metalenses based on the geometric phase compensation, the propagation phase compensation and the resonant phase compensation, respectively. The illuminating wavelength takes 488 nm, 532 nm and 633 nm, respectively. Figure 5D–F show longitudinal intensity distributions of the focusing vortex metalenses based on the three kinds of phase delays with the wavelength taking three values.

From the results in Figure 5A–C, one can see that for any kind of focusing metalens, the focusing spot appears about at the propagation distance of z = 6.3 μm for the illuminating wavelength of 488 nm, z = 5.5 μm for the wavelength of 532 nm and z = 4.5 μm for the wavelength of 633 nm. The disperse effect for three kinds of focusing metalenses are almost the same. For the same metalens, the focusing intensities for three cases are different. The focusing effect for the wavelength of 633 nm is the strongest and the focusing effect for the wavelength of 532 nm is the weakest. The difference between two cases is distinct for the resonant phase metalens. From the results of vortex metalenses shown in Figure 5D–F, one can see the similar disperse effect. The focusing effect for the wavelength of 633 nm is also the strongest and the focusing effect for the wavelength of 532 nm is also the weakest. Comparing the transverse distributions of the focusing vortices, one can see the symmetry of vortex for the geometric phase vortex metalens is the best.

The additional phase delay for any kind of metalenses depends on the incident wavelength, which can be seen from the equations in (1)–(4). The transmittance of the phase device for a different incident wavelength is different because of spectral effect of single nanounits. Therefore, the intensities for different phase devices change with the incident wavelength. With the help of phase compensation techniques, like the utilization of the special material for the nanounits with no obvious change in the wide waveband [35,36,37] or the especial structure design [38,39], it may eliminate the disperse effect of the phase metasurface device.

## 6. Conclusions

This paper analyzes the performance of phase devices including focusing metalenses and focusing vortex metalenses based on the geometric phase, the propagation phase and the resonant phase. In terms of the formation of focusing spots and focusing vortices, the design complexity of the devices, the focusing intensities, the uniformity of vortices and the polarization dependence change with the phase compensation modes. The geometric phase devices have simple designs and uniform intensity distributions, yet their working efficiencies are lower and the presented results show circular polarization dependence. The propagation and resonant phase devices have higher efficiency, yet the structure designs of the devices may be more complex and the presented results show cross circular polarization or orthogonal linear polarization conversion. The uniformity for the presented results is difficult to control. Though these phenomena only for the metalenses and vortex metalenses are presented in this paper, they have the universality for the phase devices based on three phase compensation techniques. We think this work provides the readers with the intuitive comparison of metasurface phase devices based on different phase compensation techniques and it is helpful for choosing metasurface devices suitable for the practical applications in different fields like optical communications, optical integration and quantum information processing.

## Figures and Tables

**Table 1:** Structure parameters of three metalenses with *f* = 4.5 μm.

	*Parameters*	*r*(μm)	2.93	3.85	4.66	5.4
*Shapes*						
V-shaped nanohole		*l*(nm)	113	110	148	158
		*w*(nm)	40	40	40	40
		*γ*(rad)	π	2π/3	π/2	π/3
L-shaped nanohole		*l*(nm)	190	260	260	240
		*w*(nm)	130	190	120	60
Cross nanohole		*θ*(rad)	4.31	7.05	9.82	12.55

## Data Availability

All data are contained within the article.
