# Correction: Lu et al. Performance Analysis of Metalenses Based on Three Kinds of Phase Compensation Techniques. *Nanomaterials* 2021, *11,* 2091

**DOI:** 10.3390/nano13071213

**Published:** 2023-03-30

**Authors:** Peiyao Lu, Changda Zhou, Zhen Mou, Danhua Liu, Shuyun Teng

**Affiliations:** Shandong Provincial Engineering and Technical Center of Light Manipulations & Shandong Provincial Key Laboratory of Optics and Photonic Device, School of Physics and Electronics, Shandong Normal University, Jinan 250014, China

## Text Correction

There was an error in the original publication [1].

Paragraphs 2 and 3 of Section 2 were completely repeated, and paragraph 3 must be deleted.

A correction has been made to the second section “2. Determination for the Nanounit Parameters”: paragraph 3 has been deleted.

The abbreviation for vertical linearly polarized light: “(VLP)” is missing in the text.

A correction has been made to the third section “3. Metalenses Based on Three Phase Compensation Techniques”. The content is in paragraph 4 which is included below:

In order to ensure the same transmittance, the total transmission area of nanoholes for each metalens is the same, and the transmission area of all the nanoholes at the same radius also takes the same value. Figure 2 gives the schematic diagrams for three kinds of metalenses and their focusing intensity distributions with the left-handed circularly polarized (LCP) light, right-handed circularly polarized (RCP) light, horizontal linearly polarized (HLP) light and vertical linearly polarized (VLP) light illumination. Figure 2A shows the results for the geometric phase metalens, Figure 2B gives the results for the propagation phase metalens and Figure 2C corresponds to the resonant phase metalens. For convenience comparison with respect to the intensity of the patterns in Figure 2C, the intensities in Figure 2A increase six times and the intensities in Figure 2B increase about three times. For all the structures, the nanoholes with the same parameters are on the same circles and the gap of two circles is about 1000 nm.

## Error in Table

In the original publication, there was a mistake in “Table 1” as published. “*γ*(nm)” in the second column of the fourth row of the table must be changed to “*γ*(rad)”. The corrected “Table 1” appears below:

## Error in Figure Caption

There was an error in the original publication. The figure caption for Figure 2 refers to “LCP, RCP, HCP and VLP light illumination”, and HCP here should be corrected to HLP.

A correction has been made to the third section “3. Metalenses Based on Three Phase Compensation Techniques”. The content is in the figure caption for Figure 2:

**Figure 2.** Schematic diagrams of three metalenses and their focusing intensity distributions with LCP, RCP, HLP and VLP light illumination, where (A) is for the geometric phase metalens, (B) is for the propagation phase metalens and (C) is for the resonant phase metalens. The inserted arrows in the intensity distributions denote the incident polarization states.

The authors apologize for any inconvenience caused and state that the scientific conclusions are unaffected. This correction was approved by the Academic Editor. The original publication has also been updated.

## Figures and Tables

**Table 1:** Structure parameters of three metalenses with *f* = 4.5 μm.

	*Parameters*	*r*(μm)	2.93	3.85	4.66	5.4
*Shapes*						
V-shaped nanohole		*l*(nm)	113	110	148	158
		*w*(nm)	40	40	40	40
		*γ*(rad)	π	2π/3	π/2	π/3
L-shaped nanohole		*l*(nm)	190	260	260	240
		*w*(nm)	130	190	120	60
Cross nanohole		*θ*(rad)	4.31	7.05	9.82	12.55

## References

[1] Lu P., Zhou C., Mou Z., Liu D., Teng S. (2021). Performance Analysis of Metalenses Based on Three Kinds of Phase Compensation Techniques. Nanomaterials.

